# Production of *N*^*α*^-acetylated thymosin α1 in *Escherichia coli*

**DOI:** 10.1186/1475-2859-10-26

**Published:** 2011-04-22

**Authors:** Yuantao Ren, Xueqin Yao, Hongmei Dai, Shulong Li, Hongqing Fang, Huipeng Chen, Changlin Zhou

**Affiliations:** 1 Institute of Biotechnology, Academy of Military Medical Sciences, 20 DongDa Street, FengTai District, Beijing 100071, China; 2 Institute of Neurosurgery, General Hospital of Beijing Military Command, 5 NanMenCang, Beijing 100700, China; 3 School of Life Science and Technology, China Pharmaceutical University, 24 Tong JiaXiang, Nanjing 210009, China

**Keywords:** *N*^*α*^-acetylation, thymosin α1, *Spl *DnaX intein, RimJ, protein cleavage

## Abstract

**Background:**

Thymosin α1 (Tα1), a 28-amino acid *N*^*α*^-acetylated peptide, has a powerful general immunostimulating activity. Although biosynthesis is an attractive means of large-scale manufacture, to date, Tα1 can only be chemosynthesized because of two obstacles to its biosynthesis: the difficulties in expressing small peptides and obtaining *N*^*α*^-acetylation. In this study, we describe a novel production process for *N*^*α*^-acetylated Tα1 in *Escherichia coli*.

**Results:**

To obtain recombinant *N*^*α*^-acetylated Tα1 efficiently, a fusion protein, Tα1-Intein, was constructed, in which Tα1 was fused to the N-terminus of the smallest mini-intein, *Spl *DnaX (136 amino acids long, from *Spirulina platensis*), and a His tag was added at the C-terminus. Because Tα1 was placed at the N-terminus of the Tα1-Intein fusion protein, Tα1 could be fully acetylated when the Tα1-Intein fusion protein was co-expressed with RimJ (a known prokaryotic *N*^*α*^-acetyltransferase) in *Escherichia coli*. After purification by Ni-Sepharose affinity chromatography, the Tα1-Intein fusion protein was induced by the thiols β-mercaptoethanol or d,l-dithiothreitol, or by increasing the temperature, to release Tα1 through intein-mediated N-terminal cleavage. Under the optimal conditions, more than 90% of the Tα1-Intein fusion protein was thiolyzed, and 24.5 mg Tα1 was obtained from 1 L of culture media. The purity was 98% after a series of chromatographic purification steps. The molecular weight of recombinant Tα1 was determined to be 3107.44 Da by mass spectrometry, which was nearly identical to that of the synthetic version (3107.42 Da). The whole sequence of recombinant Tα1 was identified by tandem mass spectrometry and its N-terminal serine residue was shown to be acetylated.

**Conclusions:**

The present data demonstrate that *N*^*α*^-acetylated Tα1 can be efficiently produced in recombinant *E. coli*. This bioprocess could be used as an alternative to chemosynthesis for the production of Tα1. The described methodologies may also be helpful for the biosynthesis of similar peptides.

## Background

The increasing use of peptides as pharmaceutical agents, especially in the antiviral and anti-infective therapeutic areas, requires cost-effective production on a large scale [1]. Thymosin α1 (Tα1), with its primary structure Ac-SDAAVDTSSEITTKDLKEK KEVVEEAEN, has a powerful general immunostimulating activity. Tα1 is a promising medicine and is currently used for the treatment of various bacterial infections (including antibiotic-resistant tuberculosis), viral diseases (hepatitis B and C), and as an anti-tumor agent [2].

Currently, Tα1 is either isolated from calf thymus using a multistage chromatographic purification or is obtained by chemical synthesis [3,4]. The chemosynthetic version of Tα1 has now been launched by SciClone under the trade name Zadaxin in approximately forty countries for the treatment of hepatitis B. Primary research indicates that Zadaxin may be also useful in treating a number of other diseases, including hepatitis C, non-small cell lung cancer, melanoma, and HIV/AIDS. In addition, Zadaxin is also indicated as a vaccine adjuvant, to enhance the effectiveness of the influenza and hepatitis B vaccines [5]. To reduce the production cost, a biotechnological approach using recombinant gene expression in bacteria seems promising. However, there are two main obstacles to the biosynthesis of Tα1: the bacterial expression of small peptides is difficult in general; and this protein requires N-terminal acetylation.

It is well known that direct expression of small polypeptides in *E. coli *is unsuccessful in most cases, because short peptides are often subject to degradation by proteases in the host cells, which leads to a significant decrease in yield and confers difficulties in separating the target peptides from their degraded fragments [6]. Several approaches have been taken to improve the expression of small peptides and to alleviate proteolysis, one of which is to fuse the target peptide to a carrier protein named the "fusion partner" [7,8]. The fusion partner is often highly expressed in the host cells with an affinity tag. This not only elevates the expression level of the fusion protein and thus the yield of the target peptide, but also facilitates purification of the fusion protein. One problem of this approach is the use of chemical reagents or proteases to cleave the target peptide from its fusion partner. While the use of proteases may be expensive and sometimes non-specific, chemical methods usually require harsh reaction conditions that may damage or structurally modify the target peptides, in addition to having a low specificity and reducing yield.

Fortunately, several of these problems can be solved by using intein-mediated protein purification. The intein-mediated self-cleavage system has been recently developed as a powerful tool for protein expression, purification, ligation, and amidation [9-14]. Target peptides can be synthesized with fusions that have been genetically engineered to achieve cleavage of the peptide bond at either the N- or C-terminus without the use of specific proteases or chemical reagents [9-12,14,15]. Thymosins have been successfully expressed in *E. coli *in this way [16,17]. However, the yield is still low and an *in vitro *acetylation step is required. Recently, an *in vitro *non-enzymatic acetylation of recombinant Tα1 was reported [18]. However, because in all previous cases thymosins have been fused to the C-terminus of the fusion partner, they cannot be acetylated at the N-terminus *in vivo*.

In a previous study, we revealed that N-terminal acetylation of recombinant Tα1-fused protein in *E. coli *is catalyzed by RimJ, and that fully acetylated Tα1 can be obtained by co-expressing it with RimJ [19]. This was the first description that acetylation of an ectopic protein in *E. coli *could be catalyzed by RimJ, a well-known prokaryotic N-terminal acetyltransferase. To produce Tα1, we constructed a fusion protein, named Tα1-Intein, in which Tα1 was fused to the N-terminus of a mini-intein, *Spl *DnaX intein (136 amino acids) [20]. The *Spl *DnaX intein identified in the cyanobacterium *Spirulina platensis *is the smallest thus far identified, although its splicing activity has not been experimentally proven [21]. For N-terminal cleavage, we constructed a modified *Spl *DnaX intein with a C-terminal alanine residue instead of asparagine [22]. Here, we show that this small, modified mini-intein is very suitable for N-terminal cleavage to release Tα1.

## Materials and methods

### Bacterial strains, media, and reagents

*E. coli *DH5α (F^-^, *supE*44, *ΔlacU*169 (*φ*80, *lacZΔ*M15), *hsdR*17, *recA*1, *endA*1, *gyrA*96, *thi*, *relA*1) was used for all plasmid propagation and its genome was used as a template for amplifying *rimJ*. *E. coli *BL21(DE3) (F^-^, *omp*T, *hsd*S_B_(r_B_^-^m_B_^-^), *gal*, *dcm*, (DE3)) was used for expressing proteins. Plasmids pET22b(+), pET28a(+), and pACYCDuet-1 (Novagen, EMD Chemicals, Darmstadt, Germany) were used as expression vectors. All the restriction enzymes, T4 DNA ligase, *Pyrobest*™ DNA polymerase, and DNA molecular weight marker were from TaKaRa Biotechnology (Dalian, China). Protein low molecular weight marker was purchased from GE Healthcare. Synthetic Tα1 was from SciClone Pharmaceuticals International Ltd. (Hong Kong, China). Other chemicals used in this study were of analytical or higher grade. The primers were synthesized by Shanghai Sangon Biological Engineering Technology & Services (Sangon; Shanghai, China).

### Plasmid construction

Construction of plasmid pACYC-rimJ for the expression of RimJ has been described elsewhere [19]. The gene that encodes the *Spl *DnaX intein (136 amino acid residues; from *Spirulina platensis*) was designed with the *E. coli *codon bias to ensure high expression in *E. coli*. It contains EcoRI and XhoI restriction enzyme sites. We mutated the C-terminal amino acid from Asn to Ala. To introduce an AflII site, we also mutated the third amino acid at the N-terminus from Thr to Ser. The gene was synthesized by Sangon. The gene product and vector pET22b(+) were both digested with EcoRI and XhoI. The fragments were purified after 1% agarose gel electrophoresis, and then ligated using T4 DNA ligase to yield plasmid pET-S.

Two partially complementary, single-stranded DNA oligonucleotides encoding the entire human Tα1 but with *E. coli *codon preference were synthesized. The sequences were forward primer, TA (5;-GAA TTC CAT ATG TCA GAT GCA GCA GTA GAT ACT AGC TCT GAA ATC ACT ACC AAA GAC CTG AAG GAG AAG AAG-3;) containing an NdeI site (underlined), and reverse primer, TAS (5;-GCT GCT ACT TAA GCA GTT CTC AGC CTC TTC GAC AAC TTC CTT CTT CTC CTT CAG GTC-3;) containing an AflII site (underlined). The conditions for PCR amplification were as follows: 95°C for 5 min; 30 cycles of 94°C for 30 s, 56°C for 30 s, and 72°C for 10 s, with a final hold for an extra 10 min at 72°C. The PCR product and vector pET-S were both digested with NdeI and AflII. The fragments were purified by 1% agarose gel electrophoresis, then ligated using T4 DNA ligase to generate expression plasmid pET-Tα1-Intein. Clones were selected by ampicillin screening, and then the positive clones were checked by DNA sequencing to ensure they contained the correct Tα1-Intein sequence.

### Protein expression and purification of the Tα1-Intein fusion protein and optimization of cleavage conditions

*E. coli *strain BL21 (DE3) was transformed with the plasmids pACYC-rimJ and pET-Tα1-Intein to express Tα1-Intein and RimJ. The strain was cultivated in the auto-inducible medium FML (K_2_HPO_4_•3H_2_O, 7 g/L; NaH_2_PO_4_•2H_2_O, 3 g/L; NaCl, 2.5 g/L; yeast extract, 12 g/L; tryptone, 15 g/L; MgSO_4_•7H_2_O, 0.5 g/L; glucose, 2 g/L; lactose, 0.722 g/L), containing 100 μg/ml ampicillin and 50 μg/ml chloramphenicol at 37°C, with shaking at 230 rpm for 12 h. Cells were harvested by centrifugation (5,000 × *g*, 20 min, 4°C); 10.5 g of wet cells were obtained from 1 L of culture fluid. For Tα1-Intein purification, the cell pellets were resuspended in buffer A (50 mM sodium phosphate, 0.5 M NaCl, 20 mM imidazole, pH 7.0) and lysed by sonication for 30 min (4 s working, 5 s free) in an ice-water bath. Soluble protein was separated from cell debris by centrifugation at 15,000 × *g*, 4°C for 30 min, filtered through a 0.45-μm membrane, and loaded onto a 20 ml HisTrap Fast Flow column (GE Healthcare, Fairfield, CT, USA) pre-equilibrated with buffer A, using Ni-Sepharose affinity chromatography. Bound proteins were eluted with buffer B (50 mM sodium phosphate, 0.5 M NaCl, 400 mM imidazole, pH 7.0), and then the peak fractions, including Tα1-Intein, were pooled and stored at -20°C.

The purified fusion protein Tα1-Intein was incubated with 100 mM β-mercaptoethanol (β-ME) or d,l-dithiothreitol (DTT) at different temperatures (4, 30, 37, 42, 55, or 60°C) for 24 h. As a control, purified Tα1-Intein was incubated at the same temperature for 24 h in the absence of β-ME and DTT. All reactions were terminated by mixing the protein samples with 2× sodium dodecyl sulfate (SDS)-polyacrylamide gel electrophoresis (PAGE) buffer and boiling for 3 min. The proteins were separated by 17% SDS-PAGE followed by staining with Coomassie Brilliant Blue. The stained gels were digitized with an HP4400C (Hewlett-Packard, Palo Alto, CA, USA) and the scanned images were analyzed with Gel-Pro software (Media Cybernetics, Inc., Bethesda, MD, USA). The percentage of cleavage was determined by the intensity of the precursor Tα1-Intein band of the sample compared with that of the control.

### Purification and characterization of recombinant Tα1

The solution of Tα1-Intein fusion protein was supplemented with 100 mM β-ME and incubated at 42°C for 24 h. Before centrifugation, ammonium sulfate was added to the eluate to approximately 1 M. The supernatant was adjusted to pH 7.0 and filtered through a 0.45-μm membrane, then loaded onto a Phenyl Sepharose 6 Fast Flow column (high sub) (GE Healthcare) that had been pre-equilibrated with buffer C (50 mM sodium phosphate with 1 M ammonium sulfate, pH 7.0). According to previous experiments, the Tα1 target protein was in the through fractions. To acquire highly pure Tα1, we pooled the through fractions and then applied them to a SORCE30 RPC column (GE Healthcare) that had been pre-equilibrated with buffer D (0.1% H_3_PO_4_), eluted fractions on a gradient of 100 ml, 0-100% buffer E (25% v/v acetonitrile, 0.1% H_3_PO_4_), and collected the elution fractions containing the target protein. After adjusting the pH to 7.0, the eluate was finally loaded onto a Q Sepharose Fast Flow column pre-equilibrated with buffer F (50 mM sodium phosphate, pH 7.0) followed by elution on a linear gradient of 100 ml, 0-100% buffer G (50 mM sodium phosphate, 1 M NaCl, pH 7.0). The elution fractions containing the target protein were pooled.

After this multi-stage chromatographic purification, the yield and purity of recombinant Tα1 were analyzed using reverse phase-high performance liquid chromatography (RP-HPLC) using a Hypersil 300 Å C18 5-μm column, with a 14-25% acetonitrile/0.1% H_3_PO_4 _gradient and a 1 ml/min flow rate.

The molecular mass of recombinant Tα1 was determined by quadrupole time-of-flight (Q-TOF) mass spectrometry (MS) using a Q-TOF 2 mass spectrometer (Micromass, UK) after desalting the sample on a C18 Zip-Tip (Millipore, Billerica, MA) and eluting the product in acetonitrile, water, and trifluoroacetic acid (50:50:0.5). The whole sequence of recombinant Tα1 was identified by tandem MS.

The biological activity of Tα1 was determined by E-rosette assay [23]. Briefly, fresh pig thymus lymphocytes were placed with RPMI-1640 and 100 μg/L recombinant Tα1 for 2 h at 37°C, 50 mL/L CO_2 _in a humidified incubator, using chemosynthetic Tα1-Zadaxin (SciClone Pharmaceuticals, Inc.) and Hank's buffer as parallel controls. Sheep red blood cells were then added and incubation continued for another 3 h. The percentage of E-rosette-forming cells (ERFC) was calculated by counting the number of E-rosette-forming lymphocytes out of a total of 200 lymphocytes; the experiments were repeated three times. The biological activity was defined as the percentage of ERFC in the sample minus the percentage of ERFC in the Hank's buffer control.

## Results

### Protein expression and purification of Tα1-Intein and optimization the cleavage conditions

**Figure 1:**
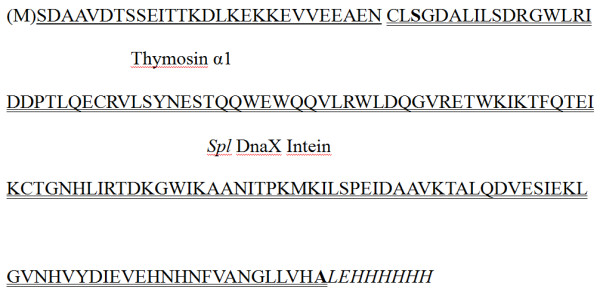
**Amino acid sequence of the fusion protein Tα1-Intein (thymosin α1-*Spl *DnaX intein)**.

**Figure 2:**
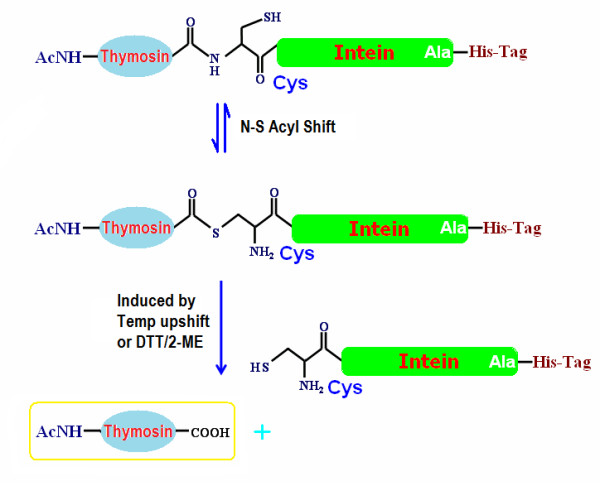
**N-terminal cleavage mediated by the modified mini-intein to release Tα1**.

**Figure 3:**
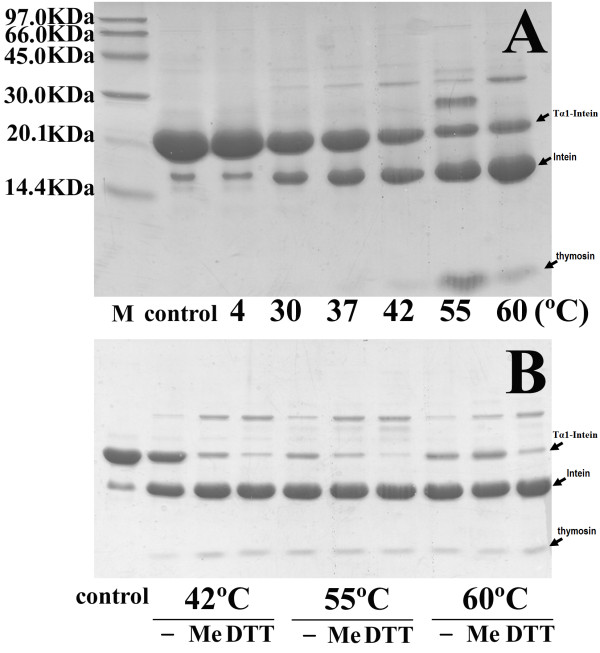
**Induction of cleavage at the N-terminus of the intein in the Tα1-Intein fusion protein**. A. Cleavage of purified Tα1-Intein fusion protein was induced at various temperatures for 24 h. "M", molecular weight marker; "control", pre-incubation sample. B. The purified Tα1-Intein fusion protein was incubated at different temperatures for 24 h. "control", pre-incubation sample; "-", without β-ME or DTT; "ME", with 100 mM β-ME; "DTT", with 100 mM DTT.

**Figure 4:**
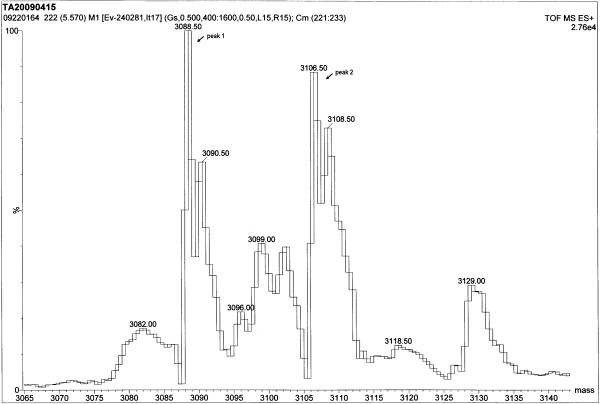
**Q-TOF-MS analysis of recombinant Tα1 released from the Tα1-Intein fusion protein by DTT-induced cleavage**. "peak 1", the dehydrated form of recombinant Tα1; "peak 2", the normal form of recombinant Tα1.

To obtain *N*^*α*^-acetylated human Tα1, Tα1-Intein and RimJ were expressed from plasmids pET-Tα1-Intein and pACYC-rimJ, respectively, in *E. coli *strain BL21 (DE3). The fusion protein Tα1-Intein (19749 Da; amino acid sequence shown in Figure 1) was purified using Ni-Sepharose affinity chromatography on a single HisTrap FF column, then treated at different temperatures with or without 100 mM β-ME or DTT. As the modified mini-intein has Ala as its C-terminal residue instead of Asn, the Tα1-Intein fusion protein can undergo N-terminal cleavage to release Tα1, as shown in Figure 2. The amount of cleavage increased with increasing temperature. At 55°C, approximately 80% of Tα1-Intein was hydrolyzed after 24 h, but at 60°C, the Tα1 band became weaker and the intein band did not (Figure 3A). This may be because of hydrolysis of Tα1. In the presence of 100 mM β-ME or 100 mM DTT, the cleavage efficiency was more than 90% at 42°C after 24 h. The efficiency of N-terminal cleavage induced by DTT was slightly higher than that induced by the same concentration of β-ME (Figure 3B). However, the molecular weight of the Tα1 obtained from thiolyzed Tα1-Intein with 100 mM DTT was not uniform. A spurious peak, 18 Da smaller than the calculated value (see peaks 1 and 2 in Figure 4) was observed. The whole sequence of peak 1 was identified by Q-TOF MS/MS and its C-terminal Asn residue was shown to be dehydrated (data not shown). Considering the stability and cleavage efficiency of the Tα1-Intein fusion protein, the optimal cleavage conditions were established to be 100 mM β-ME at 42°C for 24 h.

### Purification and characterization of recombinant Tα1

**Figure 5:**
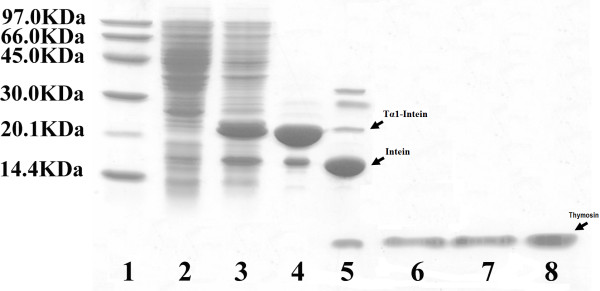
**SDS-PAGE of Tα1 expression and purification**. Lane 1: molecular weight marker; Lane 2: non-induced bacterial lysate cultured in LB medium; Lane 3: induced bacterial lysate cultured in FML medium; Lane 4: Tα1-Intein purified by Ni-Sepharose; Lane 5: Tα1-Intein incubated with 100 mM β-mercaptoethanol (ME) at 42°C for 24 h; Lane 6: combined through fractions, after Phenyl Sepharose 6 Fast Flow column; Lane 7: combined eluted fractions, after SORCE30 RPC column; Lane 8: combined eluted fractions, after Q Sepharose Fast Flow column.

Recombinant *N*^*α*^-acetylated Tα1 was purified from a 1-L shaking flask culture of bacteria as described above (Figure 5). The final amount and the purity of Tα1 were 24.5 mg and 98%, respectively, as determined by RP-HPLC.

**Figure 6:**
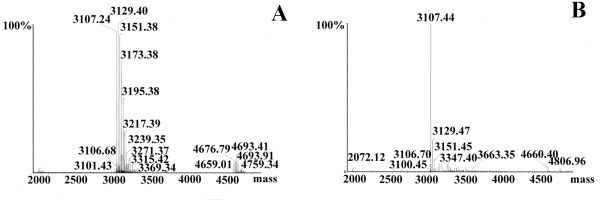
**Q-TOF MS analysis of the synthetic Tα1 standard (A) and recombinant Tα1 (B)**. Apart from the peak at 3107 Da, there are peaks representing several sodium adducts at 3129, 3151, and 3173 Da.

We mixed the synthetic Tα1 standard and the recombinant Tα1, and analyzed the mixture using RP-HPLC. Because there was only one peak, this indicated that the HPLC retention time of recombinant Tα1 was identical to that of the synthetic Tα1 standard (data not shown). The molecular weight of synthetic Tα1 (3107.42 Da; Figure 6A) was determined by MS to be almost identical to that of recombinant Tα1 (3107.44 Da; Figure 6B). The Q-TOF MS spectra revealed several sodium adducts (peaks at 3129, 3151, and 3173 Da; Figure 6A, B). Instead of the sample molecules being ionized by the addition of a proton, H^+^, the molecules in these peaks were ionized by the addition of one or more sodium cations, Na^+^. Moreover, there was no peak corresponding to non-acetylated Tα1 in Figure 4B, so the N-terminal Ser of recombinant Tα1 was fully acetylated; this was confirmed by Q-TOF MS/MS (data not shown). These results suggest that intein-mediated protein cleavage occurred specifically at the expected site, and that recombinant Tα1 was *N*^*α*^-acetylated on its native primary amino acid sequence.

The percentage of ERFC was calculated by counting the number of E-rosette-forming lymphocytes out of a total of 200 lymphocytes [23]. Recombinant Tα1 and chemosynthetic Tα1 demonstrated a similar ability to increase the percentage of ERFC (16.1 ± 3.3% (*n *= 3)) relative to the number formed in the Hank's buffer control.

## Discussion

Although the requirement for Tα1 for clinical applications is increasing, Tα1 is still mainly obtained by chemical synthesis. While chemically synthesized Tα1 can reach a high level of purity, it is necessary to eliminate side products at each step, including those with incorrect joining and dextral compounds. Moreover, the longer the peptide, the more intricate the chemical synthesis process becomes. Tα1 comprises 28 amino acids, and its chemical synthesis is expensive. Therefore, a biosynthetic method using recombinant gene expression in bacteria is desirable. Here, we expressed fully *N*^*α*^-acetylated Tα1 in *E. coli *using an intein fusion and co-expression with RimJ. We thus overcame the two common problems of *E. coli *polypeptide expression: the difficulties in expressing small peptides and obtaining *N*^*α*^-acetylation.

Self-splicing intein can be mutated at the N- or C-terminal splice junction to yield self-cleaving inteins, which can then be used as self-cleaving purification tags. These modified inteins can be split into two categories: C-terminal fusion-mediated cleavage can be induced by thiol reagents and pH, whereas N-terminal fusion-mediated cleavage can only be thiol-induced [10]. The expression of intein-fused thymosins has been previously reported [16,17]; however, these C-terminal cleavage methods suffer from a low yield and a lack of *in vivo *acetylation. For efficient expression of the small peptide Tα1, we chose the smallest mini-intein, *Spl *DnaX intein, consisting of 136 amino acids, as the fusion partner. As an *N*^*α*^-acetylated peptide, Tα1 must be located at the N-terminus of the fusion protein (Figure 1). Therefore, we modified the C-terminal residue of the mini-intein from Asn to Ala. *In vivo N*^*α*^-acetylation at the N-terminal Ser of Tα1 was achieved by co-expression with RimJ [19].

**Figure 7:**
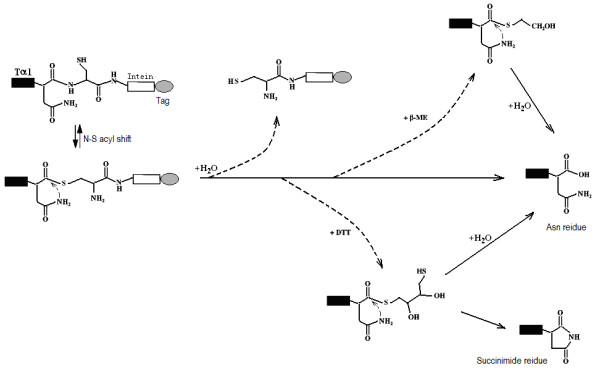
**Succinimide residue formation at the C-terminus of Tα1 during thiolysis**.

This modified mini-intein-mediated N-terminal cleavage reaction was induced by the addition of thiol reagents and/or increasing the temperature; both factors worked cooperatively. Approximately 80% of Tα1-Intein was hydrolyzed at 55°C after 24 h, but in the presence of 100 mM β-ME or DTT, the extent of N-terminal cleavage was more than 90% at 42°C after 24 h. Because the target peptide Tα1 is very stable even at 80°C [4], the cleavage conditions are compatible with the product. Although DTT induction was slightly more efficient than β-ME induction, a proportion of the released Tα1 was dehydrated (peak 1 in Figure 4) at the C-terminal Asn (data not shown), a phenomenon that has not been previously reported. One possible mechanism for this is the formation of a succinimide residue followed by nucleophilic attack of the side-chain N atom of the Asn residue during thiolysis (Figure 7). The thioester bond of Asn with DTT may more easily induce succinimide formation than that with β-ME.

The small size of the *Spl *DnaX mini-intein (136 amino acids) and its high level of expression in *E. coli *made it an attractive choice for these experiments. However, *in vivo *self-cleavage still reduced the yield of the target peptide Tα1. A new methodology involving deletion of the glutathione synthetase gene (*gshA*) has been reported to overcome this limitation [24]. However, in this study, deletion of *gshA *did not have a significant effect on the *in vivo *self-cleavage of the Tα1-Intein fusion protein (data not shown). Even with this limitation, we were able to obtain 24.5 mg of Tα1 from 1 L of culture media in a shaking flask, and the purity was more than 98% after a series of chromatographic purification steps. Furthermore, the biological activity of the recombinant Tα1 (16.1 ± 3.3%) exceeded the 10% minimum for a usable product as defined by the Chinese Pharmacopoeia; recombinant Tα1 is deemed to be active in enhancing the formation of T-cell receptors in sheep erythrocytes.

## Conclusions

In this study, we described an efficient procedure for the production of large quantities of recombinant *N*^*α*^-acetylated Tα1 in *E. coli *by combining N-terminal cleavage technology mediated by fusion with the smallest mini-intein, *Spl *DnaX, and co-expression with RimJ. The method is simple and cost-effective, and presents an attractive alternative to chemosynthetic approaches. The methodologies described here may also be helpful in the biosynthesis of similar peptides.

## Competing interests

The authors declare that they have no competing interests.

## Authors' contributions

YR co-designed and performed all the experiments, and analyzed the data. XY participated in design of all the experiments, analyzed the data and drafted the manuscript. HF initiated and coordinated the study, and contributed to the experimental design, data interpretation, and reviewing the manuscript. HD participated in the bacterial culture, protein purification, and SDS-PAGE. SL assisted with the protein purification. HC and CZ helped to coordinate the study. All authors have read and approved the final manuscript.
